# Underdocumentation of laboratory-defined acute kidney injury in internal medicine wards: a retrospective cohort study

**DOI:** 10.1080/0886022X.2026.2723684

**Published:** 2026-09-06

**Authors:** Nomy Levin-Iaina, Sharif Zalum, Mustafa Seh, Elena Rotshild, Rey Biton

**Affiliations:** ^a^ Department of Nephrology and Hypertension, Barzilai University Medical Center, Ashkelon, Israel; ^b^ School of Medicine, Faculty of Health Sciences, Ben Gurion University of the Negev, Beer Sheva, Israel; ^c^ Department of Anesthesiology, Barzilai University Medical Center, Ashkelon, Israel; ^d^ Internal Medicine Department D, Barzilai University Medical Center, Ashkelon, Israel

**Keywords:** Acute kidney injury, underdocumentation, clinical recognition, internal medicine

## Abstract

Acute kidney injury (AKI) is common among hospitalized patients, but laboratory-defined AKI may not be documented. We aimed to determine the frequency of undocumented AKI in internal medicine wards, compare clinical characteristics and in-hospital outcomes by documentation status, and explore associated factors. We conducted a retrospective single-center study of adult hospitalizations with AKI defined by serum creatinine criteria; urine-output criteria were unavailable. Because reliable pre-admission creatinine values were not consistently available, the lower of admission and discharge creatinine was used as reference and compared with maximal creatinine. Among 161 hospitalizations in 161 unique patients, 39 (24.2%) were undocumented. Undocumented cases had lower admission creatinine (2.1 vs 2.9 mg/dL; *p* < 0.001), lower maximal creatinine (2.2 vs 3.3 mg/dL; *p* < 0.001), and more frequent malignancy (23.1% vs 7.4%; *p* = 0.016). Length of stay and in-hospital mortality did not differ significantly. In 149 hospitalizations with a demonstrable creatinine change, 33 (22.1%) were undocumented. Higher maximal creatinine remained associated with lower odds of underdocumentation (adjusted OR 0.67 per 1 mg/dL, 95% CI 0.50–0.89; *p* = 0.006). Approximately one quarter of serum creatinine-defined AKI cases were undocumented, particularly those with lower maximal creatinine. Prospective studies should assess whether structured AKI detection and documentation improve kidney-related care and outcomes.

## PLAIN LANGUAGE SUMMARY

Acute kidney injury is a sudden worsening of kidney function that is common in hospitalized patients. It is usually identified by changes in blood creatinine levels, but the diagnosis may not always be clearly documented in the medical record.

We reviewed 161 patients with laboratory-defined acute kidney injury who were hospitalized in internal medicine wards. In 39 patients, approximately one in four cases, acute kidney injury was not documented or clearly recognized in the medical record. Undocumented cases generally had lower creatinine values and more often remained at a lower maximum AKI stage during hospitalization.

These findings suggest that less marked but potentially clinically relevant changes in kidney function may be less likely to be documented than more obvious abnormalities. The study did not evaluate whether lack of documentation affected treatment, follow-up, or long-term outcomes. Future studies should examine whether structured electronic detection and documentation systems can improve AKI recognition, kidney-related care processes, and follow-up.

## KEY MESSAGES

**What is known:** Laboratory-defined acute kidney injury may not be explicitly documented or clinically recognized, but most previous studies have focused on critically ill populations rather than patients in general internal medicine wards.

**What this study adds:** In this single-center cohort, approximately one quarter of serum creatinine-defined AKI cases were undocumented. Undocumented cases had lower admission and maximal creatinine values, with lower maximal creatinine representing the most consistent adjusted finding.

**Potential impact:** These findings support prospective evaluation of structured AKI detection and documentation strategies designed to identify less marked creatinine changes and determine whether they improve documentation and kidney-related care processes.

## Introduction

Acute kidney injury (AKI) is a frequent complication among hospitalized patients and is consistently associated with increased morbidity, mortality, length of stay, need for kidney replacement therapy (KRT), and subsequent chronic kidney disease [1–6]. The clinical importance of AKI extends beyond severe cases requiring dialysis. Even small or transient increases in serum creatinine have been associated with worse short- and long-term outcomes [7–9].

According to the Kidney Disease: Improving Global Outcomes (KDIGO) criteria, AKI is defined by an increase in serum creatinine of at least 0.3 mg/dL within 48 h, an increase to at least 1.5 times baseline within the preceding 7 days, or a reduction in urine output to less than 0.5 mL/kg/h for at least 6 h [2]. In the present study, AKI was assessed using serum creatinine criteria only because urine-output data were not systematically available. In routine clinical practice, serum creatinine is widely available and is often the main marker used to identify AKI outside intensive care settings. However, the availability of laboratory data does not necessarily ensure documented clinical recognition. A patient may meet biochemical criteria for AKI without the diagnosis being documented in the medical record, discharge summary, or treatment plan.

Failure to document AKI or evidence of its clinical recognition may have practical consequences, although absence of documentation does not necessarily establish that the treating clinicians were unaware of the creatinine change. Diagnosis should prompt assessment for reversible causes, medication dose adjustment, avoidance of nephrotoxic agents, evaluation of volume status, monitoring of kidney function, and post-discharge follow-up planning. From a nephrology perspective, explicit AKI documentation may facilitate linkage of the acute hospitalization event to renal recovery assessment, discharge planning, and decisions regarding post-discharge kidney function monitoring or nephrology referral.

Previous studies of AKI underrecognition have focused mainly on critically ill populations, including older patients with severe COVID-19 or invasive mechanical ventilation, in whom AKI was frequently unrecognized and associated with worse outcomes [10,11].

However, AKI is also highly relevant in general medical populations. Retrospective studies of multimorbid medical hospitalizations and older adults admitted with acute medical illness have demonstrated a substantial burden of AKI and its association with frailty, increased healthcare utilization, prolonged hospitalization, and mortality [12,13]. Less is known about AKI underdocumentation specifically in internal medicine wards, where patients are often older, multimorbid, and hospitalized for complex non-nephrological conditions.

The extent to which laboratory-defined AKI is translated into documented clinical recognition in internal medicine wards remains insufficiently characterized. This setting is distinct from the intensive care environment: patients are often admitted for diverse non-nephrological conditions, laboratory monitoring may be less intensive, and modest creatinine changes may compete with other acute clinical priorities. Therefore, evaluating AKI documentation in this population may identify specific clinical contexts in which kidney injury is at risk of being overlooked.

We therefore conducted a retrospective study of patients with serum creatinine-defined AKI identified during hospitalization in internal medicine wards at Barzilai University Medical Center. We aimed to determine the proportion of AKI cases without an explicit documented diagnosis or documented evidence of clinical recognition, compare clinical characteristics and in-hospital outcomes between documented and undocumented AKI, and identify factors associated with underdocumentation. We hypothesized that undocumented AKI would be more common in cases with lower absolute creatinine values and in clinical contexts where kidney injury may be perceived as secondary to the primary reason for admission.

## Patients and methods

### Study design and setting

We conducted a retrospective single-center observational study at Barzilai University Medical Center, a university-affiliated public hospital in southern Israel. The study included adult patients hospitalized in the internal medicine wards during July-December 2024. During the study period, adult internal medicine inpatient care was provided across five internal medicine wards within the same Internal Medicine Division. The wards operated under a broadly similar organizational model, with comparable physician and nursing staffing structures, attending supervision, daily ward rounds, admission and discharge procedures, and use of the same institutional electronic medical record and documentation system. Although individual clinicians and team composition varied over time, the general clinical workflow and documentation processes were similar across the five wards. The study was approved by the Institutional Review Board of Barzilai University Medical Center (approval no. BRZ-0026-24 from February 9, 2026). The requirement for informed consent was waived by the ethics committee due to the retrospective design and the use of anonymized data.

### Study population

All adult patients aged 18 years or older who were hospitalized in internal medicine wards during the study period and met the study’s serum creatinine-based AKI criteria were eligible for inclusion. AKI cases were identified from institutional clinical databases and verified through review of available laboratory results and medical records. Patients were included regardless of whether AKI was already present at admission or developed during hospitalization. Patients with insufficient serum creatinine measurements, maintenance dialysis, or a kidney transplant were excluded.

After eligible AKI cases were identified, patients were classified according to AKI documentation status, as detailed below. Each patient contributed only one hospitalization to the study cohort; no patient had more than one eligible hospitalization during the study period.

### AKI definition

AKI cases were initially identified from the source clinical dataset and verified through review of available laboratory results and medical records. AKI was defined using serum creatinine-based criteria only, because urine-output data were not systematically available. For each hospitalization, the analytic dataset retained three summary serum creatinine values: serum creatinine at admission, the maximal serum creatinine recorded during hospitalization, and serum creatinine at discharge. Reliable pre-admission outpatient creatinine values were not consistently available, and admission creatinine was therefore not assumed to represent stable baseline kidney function.

The available reference creatinine was defined as the lower of the admission and discharge serum creatinine values. Maximal serum creatinine was compared with this reference. We use the term ‘creatinine magnitude thresholds’ to describe a maximal-to-reference increase of at least 0.3 mg/dL and/or a maximal creatinine value at least 1.5 times the available reference. Meeting either threshold indicated a demonstrable dynamic change in serum creatinine during hospitalization.

Because the temporal sequence of all creatinine measurements and reliable pre-admission values were unavailable, this approach could not confirm the temporal components of the KDIGO definition or distinguish AKI present at admission with subsequent recovery from AKI developing after admission. We therefore refer to these events as serum creatinine-defined AKI identified during hospitalization.

AKI severity was staged using the same available reference creatinine. Stage 1 was defined as an increase in serum creatinine of at least 0.3 mg/dL or to 1.5–1.9 times the available reference; stage 2 as an increase to 2.0–2.9 times the available reference; and stage 3 as an increase to at least 3.0 times the available reference, a maximal serum creatinine of at least 4.0 mg/dL, or initiation of KRT. AKI stage represented the highest serum creatinine-based KDIGO stage reached during hospitalization and did not represent the stage at first biochemical detection or first clinical documentation.

In 12 cases identified as AKI during the original source-data review, the event could not be independently reconstructed from the three retained summary creatinine values. These cases were included in the primary cohort but excluded from the *post hoc* sensitivity analysis restricted to cases with demonstrable creatinine dynamics.

### Assessment of AKI recognition and documentation

After serum creatinine-defined AKI cases were identified, the medical records were reviewed to determine whether AKI had been documented or clinically recognized during hospitalization. Documentation was assessed using a predefined chart-review protocol that included admission notes, daily progress notes, problem lists, diagnosis lists, nephrology consultation notes when available, discharge summaries, and admission and discharge diagnoses.

AKI was considered documented or clinically recognized when the medical record contained at least one of the following: (1) explicit diagnostic terminology, including ‘AKI’, ‘acute kidney injury’, ‘acute renal failure’, or ‘acute renal insufficiency’; (2) an equivalent narrative statement clearly characterizing the change in kidney function as acute, such as ‘acute deterioration in kidney function’, ‘worsening renal function’, or ‘acute rise in creatinine’; or (3) an AKI-specific assessment or management plan explicitly linked to the acute renal change.

Such plans included renal function monitoring because of an acute creatinine rise, discontinuation or dose adjustment of nephrotoxic or renally cleared medications because of acute renal deterioration, volume management for suspected pre-renal AKI, evaluation for urinary obstruction, or nephrology consultation for AKI.

An isolated abnormal creatinine value, a nonspecific statement that creatinine was elevated without characterization of an acute change, or a generic reference to chronic renal dysfunction was not considered sufficient evidence of AKI documentation or clinical recognition. Similarly, medication changes were not considered evidence of AKI recognition unless they were explicitly linked to AKI, an acute creatinine rise, or acute deterioration in kidney function.

Cases were classified as undocumented AKI when none of these criteria were identified. Accordingly, “documented AKI” in this study refers to either explicit diagnostic labeling or documented clinical recognition and should not be interpreted as formal diagnostic coding alone.

Documentation status reflected whether AKI was documented or clinically recognized at any point during hospitalization. The original abstraction did not retain the date and time of the first creatinine measurement meeting AKI criteria or the first AKI-related documentation. Therefore, the interval between biochemical AKI onset and documentation, and the AKI stage at first documentation, could not be assessed.

Chart review was performed by one investigator using a predefined protocol. The primary reviewer had access to the complete medical record, including clinical notes, serial laboratory measurements, documented comorbidities, treatments, and the clinical course of hospitalization. Fifteen cases (9.3%) in which documentation status remained uncertain underwent additional adjudication by a senior nephrologist. For these cases, the senior nephrologist reviewed the relevant clinical documentation, serial creatinine measurements, and comorbidities but was not provided with the derived AKI stage or the predefined analytic study outcomes, including length of stay, in-hospital mortality, KRT-related outcomes, and discharge destination.

A second independent review of the full cohort or a randomly selected subset was not feasible; therefore, formal inter-rater agreement, such as Cohen’s kappa, could not be assessed. This classification reflects evidence recorded in the medical record. Absence of documented recognition does not prove that clinicians were unaware of the creatinine change, and the presence of an AKI diagnosis or documented AKI-directed action does not establish that evaluation or management was complete, appropriate, or timely.

### Data collection

Data were extracted from the electronic medical record and included demographic characteristics, comorbidities, kidney function measures, admission vital signs, primary hospitalization indication, retrospectively assigned AKI etiology, and in-hospital outcomes. Comorbidities included diabetes mellitus, hypertension, dyslipidemia, congestive heart failure, and malignancy.

Ward assignment, treating-team identifiers, clinician-level staffing characteristics, trainee composition, and nephrology consultation frequency were not retained in the analytic dataset. Therefore, ward- or team-specific documentation rates and within-ward clustering could not be evaluated.

Kidney function variables included admission serum creatinine, maximal serum creatinine during hospitalization, and serum creatinine at discharge. Admission creatinine was analyzed as a presentation value and was not considered a confirmed pre-illness baseline. The total number of creatinine measurements, intervals between measurements, and exact timing of AKI onset were not retained. Serum creatinine testing was clinician-driven rather than protocolized; therefore, monitoring frequency could not be compared between documentation groups. Preexisting chronic kidney disease, pre-admission outpatient creatinine, and pre-admission eGFR were not consistently available and were not included as study variables.

Primary hospitalization indications were classified into predefined broad clinical categories based on the admission note, emergency department assessment, and early hospitalization records. Categories included infectious disease including sepsis, cardiovascular disease, neurologic disease, pulmonary disease, gastrointestinal disease, renal disease, hematology or oncology disease, endocrine or diabetes-related complications, shock, and other indications. Only broad predefined hospitalization-indication categories were retained; specific diagnoses and cardiorenal or hepatorenal syndromes were not separately abstracted.

AKI etiology was assigned retrospectively using a predefined clinical framework based on available medical record, including the clinical course, hemodynamic and volume status, infection or sepsis, medication exposure when documented, evaluation for urinary obstruction, nephrology consultation notes, laboratory trends, and imaging when available.

Etiology categories included pre-renal, post-renal, glomerular disease, acute tubular necrosis, interstitial disease, vascular disease, and other or unspecified. When the available information was insufficient to assign a specific cause, AKI was classified as other or unspecified. Because hospitalization indication and AKI etiology were determined retrospectively, these classifications were considered descriptive and exploratory.

### Outcomes

The primary outcome was undocumented AKI, defined as the absence of either explicit diagnostic documentation or documented evidence of clinical recognition of an acute deterioration in kidney function among patients with serum creatinine-defined AKI. This outcome measured evidence recorded in the medical record and was not intended to establish clinicians’ unrecorded awareness or the adequacy of AKI management. Secondary analyses compared baseline characteristics, comorbidities, hospitalization indications, AKI etiology, creatinine profile, length of hospital stay, requirement for KRT during hospitalization, discharge with ongoing KRT, in-hospital mortality, and discharge destination according to AKI documentation status.

Data on AKI-related care processes, including renal follow-up recommendations in the discharge summary, planned repeat serum creatinine testing, nephrology referral, medication adjustment, and discontinuation or avoidance of nephrotoxic medications, were not systematically collected and therefore were not analyzed.

Discharge destination was summarized among patients discharged alive. Because several destination categories contained very small numbers, these comparisons were considered descriptive and no formal between-group significance test was reported.

### Statistical analysis

Continuous variables were assessed for distribution and are presented as medians with interquartile ranges (IQRs), and categorical variables are presented as counts and percentages. Comparisons between documented and undocumented AKI groups were performed using the Mann-Whitney U test for continuous variables and the chi-square test or Fisher’s exact test for categorical variables, as appropriate. A two-sided p-value < 0.05 was considered statistically significant.

Logistic regression was used to explore factors associated with undocumented AKI. The dependent variable was undocumented AKI, with documented AKI as the reference group. Given the limited number of undocumented events, the models were intentionally parsimonious and were considered exploratory and hypothesis-generating. The primary model included malignancy and maximal serum creatinine, selected based on the clinical relevance and associations with documentation status in univariable analyses. A secondary expanded model additionally included sex, diabetes mellitus, and congestive heart failure. The primary and expanded models contained 19.5 and 7.8 undocumented events per predictor, respectively; the expanded model was therefore considered a secondary robustness analysis rather than a definitive fully adjusted model. Model assumptions and influence were assessed using variance inflation factors, the Box-Tidwell test, Hosmer-Lemeshow testing, Cook’s distance, and leave-one-out analyses.

Because reliable pre-admission kidney function data were not consistently available, AKI staging was based on the available admission, maximal, and discharge creatinine values. The models could not adjust for preexisting CKD or pre-admission eGFR.

A *post hoc* sensitivity analysis was performed in the subgroup with demonstrable creatinine dynamics, defined as a maximal-to-reference increase of at least 0.3 mg/dL and/or a maximal creatinine value at least 1.5 times the available reference. Between-group comparisons and the exploratory logistic regression models were repeated in this restricted cohort. Cases in which AKI could not be independently reconstructed from the retained summary creatinine values were excluded. Because the available reference could be either the admission or discharge creatinine, this analysis confirmed a dynamic creatinine change during hospitalization but could not determine whether AKI was present at admission or developed after admission.

Data completeness was assessed for all variables included in the analytic dataset. Discharge creatinine was unavailable for one patient; all other variables included in the descriptive comparisons and regression models were complete. Therefore, all 161 hospitalizations contributed to the full-cohort analyses, and no imputation was required. Variables that were not systematically collected, including pre-admission outpatient creatinine, pre-admission eGFR, and urine-output data, were not included as study variables.

Because the overall distribution of hospitalization indications was not statistically significant and several categories contained small numbers, category-specific comparisons were considered *post hoc* and exploratory. These p-values were not adjusted for multiple comparisons and were interpreted descriptively. Analyses were performed using Python.

## Results

### Study population and AKI documentation status

**Figure 1.**
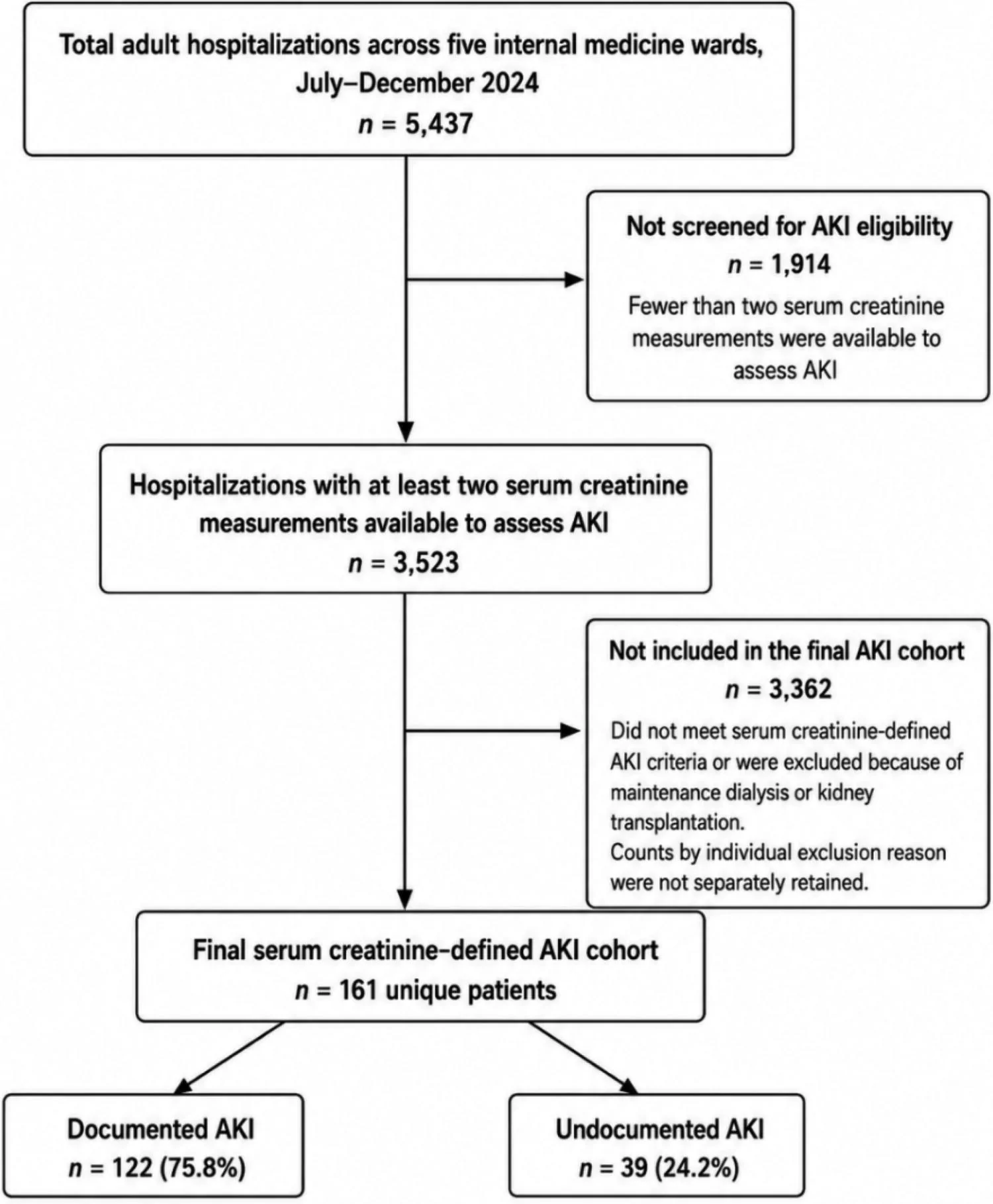
Flow diagram of study cohort identification. Of 5,437 adult hospitalizations during July-December 2024, 3,523 had at least two serum creatinine measurements available for AKI assessment. After application of the study criteria and predefined exclusions, 161 unique patients with serum creatinine-defined AKI were included, comprising 122 documented and 39 undocumented cases. Counts by individual exclusion reason were not separately retained.

During the study period, 5,437 adult hospitalizations occurred across the five internal medicine wards. Of these, 3,523 hospitalizations had at least two serum creatinine measurements available to assess AKI eligibility. Following application of the serum creatinine-defined AKI criteria and the predefined exclusion criteria, 161 hospitalizations corresponding to 161 unique patients were included in the final cohort. Of these, 122 cases (75.8%) were classified as documented AKI and 39 cases (24.2%) as undocumented AKI (Figure 1).

Because reliable pre-admission creatinine values were not consistently available, the cohort may have included both AKI present at admission with subsequent evolution or recovery and AKI developing after admission.

Creatinine dynamics could be reconstructed from the retained admission, maximal, and discharge values in 149 of 161 hospitalizations (92.5%). All 149 met the absolute ≥0.3 mg/dL threshold; 121 also met the ≥1.5-fold threshold, whereas 28 met the absolute threshold alone. No hospitalization met the relative threshold alone. The remaining 12 cases were retained in the primary analysis but excluded from the sensitivity analysis because the AKI event could not be independently reconstructed from the three retained summary creatinine values. The timing of AKI onset and the intensity of creatinine monitoring could not be compared between documentation groups because measurement timestamps and the total number of creatinine tests per hospitalization were unavailable.

#### Data completeness

No item-level data were missing for variables included in the regression models. Discharge creatinine was unavailable for one patient; all other variables included in the descriptive analyses were complete. Accordingly, all 161 hospitalizations contributed to the regression models and to analyses not involving discharge creatinine, whereas the discharge-creatinine comparison included 160 hospitalizations.

### Baseline characteristics

Baseline characteristics are presented in Table 1. The groups were similar with respect to sex distribution, admission systolic and diastolic blood pressure, heart rate, and most comorbidities, including diabetes mellitus, hypertension, dyslipidemia, and congestive heart failure.

**Table 1.** Baseline characteristics of patients with AKI according to documentation status during hospitalization in internal medicine wards.

Variable	Documented (*n* = 122)	Undocumented (*n* = 39)	p-value
**Demographics**			
Female sex, n (%)	55 (45.1%)	22 (56.4%)	0.218
Male sex, n (%)	67 (54.9%)	17 (43.6%)	
**Clinical Parameters at Admission**			
Systolic blood pressure, mmHg, median [IQR]	120.0 [103.5–145.0]	123.0 [106.0–138.5]	0.871
Diastolic blood pressure, mmHg, median [IQR]	65.0 [55.2–77.0]	70.0 [55.0–76.5]	0.776
Heart rate, beats/min, median [IQR]	82.5 [71.0–97.0]	80.0 [72.0–105.0]	0.890
**Kidney Function**			
Admission creatinine, mg/dL, median [IQR]	2.9 [2.1–4.5]	2.1 [1.5–2.8]	<0.001
**Comorbidities**			
Diabetes mellitus, n (%)	63 (51.6%)	20 (51.3%)	0.969
Hypertension, n (%)	94 (77.0%)	29 (74.4%)	0.731
Dyslipidemia, n (%)	54 (44.3%)	20 (51.3%)	0.444
Congestive heart failure, n (%)	31 (25.4%)	15 (38.5%)	0.116
Malignancy, n (%)	9 (7.4%)	9 (23.1%)	0.016

A two-sided p-value < 0.05 was considered statistically significant.

Patients with undocumented AKI had lower admission serum creatinine than those with documented AKI (2.1 mg/dL [IQR 1.5–2.8] vs. 2.9 mg/dL [IQR 2.1–4.5]; *p* < 0.001). Malignancy was more frequent in the undocumented group, occurring in 9 of 39 patients (23.1%) compared with 9 of 122 patients (7.4%) in the documented group (*p* = 0.016).

### Hospitalization indications and AKI etiology

**Figure 2.**
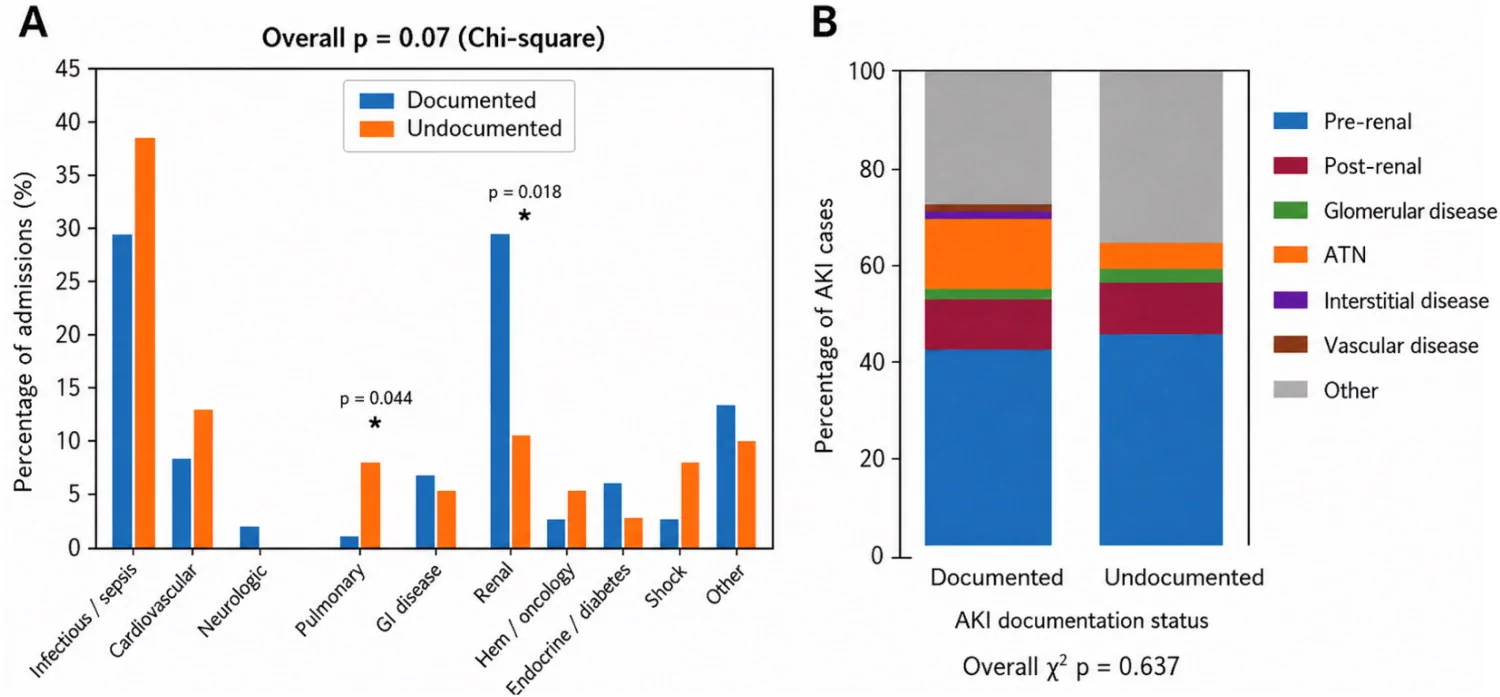
Hospitalization indications and AKI etiologies according to AKI documentation status. (A) Distribution of hospitalization indications among patients with documented and undocumented AKI. The overall between-group comparison was not statistically significant. Category-specific comparisons were *post hoc*, unadjusted for multiple comparisons, and should be interpreted as exploratory. (B) Distribution of retrospectively assigned AKI etiologies according to documentation status. Etiologic classification was based on available chart data and should be interpreted as descriptive; the overall between-group comparison was not statistically significant.

The overall distribution of hospitalization indications did not differ significantly between groups (*p* = 0.070). Given the non-significant overall comparison and the small numbers in several categories, category-specific comparisons were considered *post hoc* and exploratory. Pulmonary admissions were nominally more frequent among patients with undocumented AKI than among those with documented AKI (3/39, 7.7% vs. 1/122, 0.8%; *p* = 0.044), whereas renal admissions were nominally more frequent among patients with documented AKI (36/122, 29.5% vs. 4/39, 10.3%; *p* = 0.018) (Figure 2A). These p-values were unadjusted for multiple comparisons and should not be interpreted as confirmatory. Only broad hospitalization-indication categories were retained; therefore, the observed patterns cannot be attributed to specific admitting diagnoses.

AKI etiology was analyzed descriptively because etiologic classification was based on retrospective chart review. The overall distribution of AKI etiologies did not differ significantly between groups (*p* = 0.637). Pre-renal AKI was the most frequent etiology in both groups, occurring in 18 of 39 undocumented cases (46.2%) and 52 of 122 documented cases (42.6%). Acute tubular necrosis was numerically more frequent among documented cases, whereas other or unspecified etiologies were numerically more frequent among undocumented cases; however, these differences were not statistically significant (Figure 2B).

### Creatinine profile, AKI severity, and in-hospital outcomes according to documentation status

**Figure 3.**
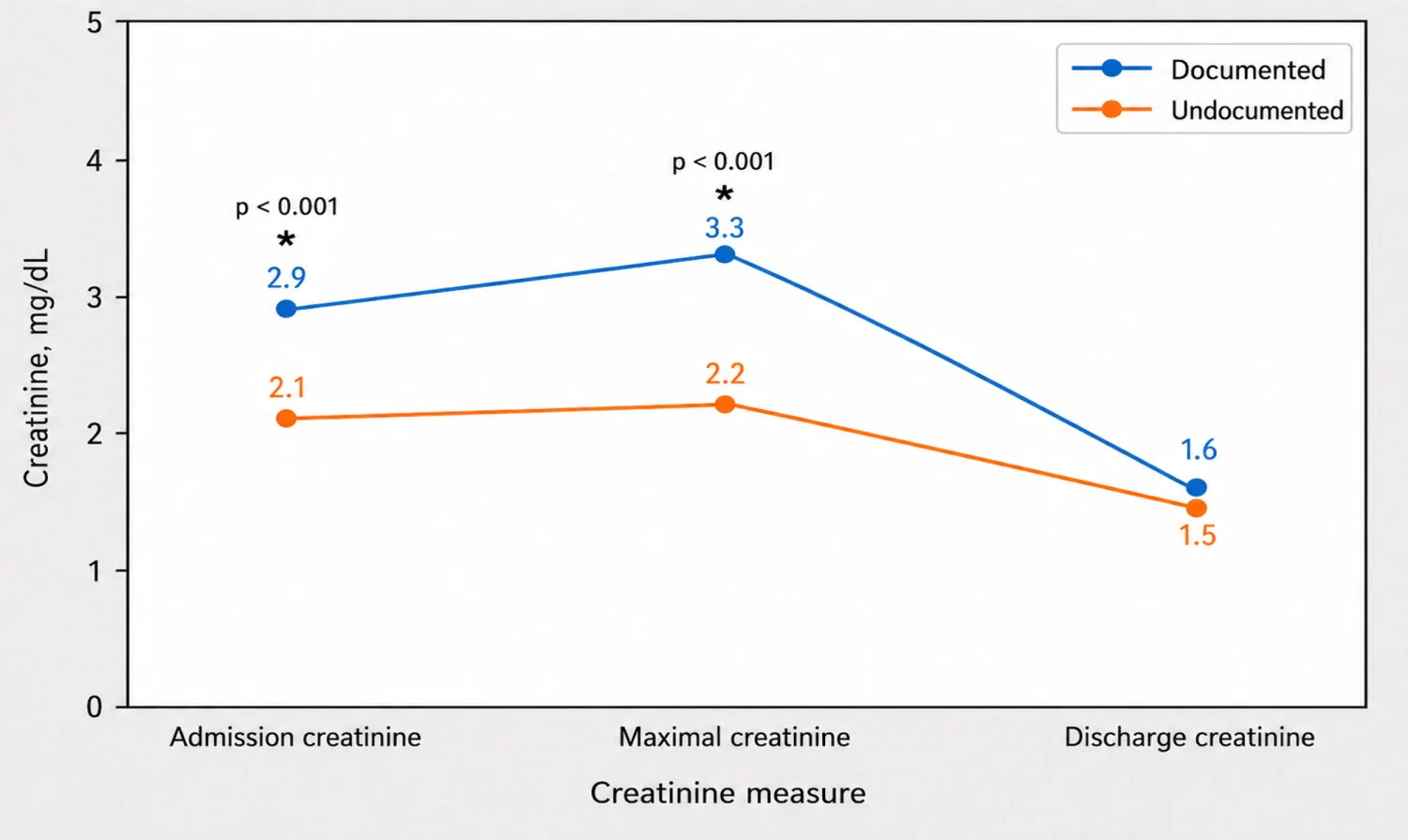
Admission, maximal, and discharge serum creatinine values among patients with documented and undocumented AKI during hospitalization in internal medicine wards. Values represent median serum creatinine levels in mg/dL. Patients with undocumented AKI had significantly lower admission creatinine and maximal creatinine compared with patients with documented AKI (both *p* < 0.001). Discharge creatinine did not differ significantly between groups. *p < 0.05 for between-group comparison at each time point.

AKI course and in-hospital outcomes are shown in Table 2. Patients with undocumented AKI had significantly lower maximal serum creatinine during hospitalization than those with documented AKI, whereas discharge creatinine did not differ significantly between groups. Serum creatinine values according to AKI documentation status are shown in Figure 3.

**Table 2.** Creatinine trajectory, AKI severity, and in-hospital outcomes according to AKI documentation status.

Variable	Documented (*n* = 122)	Undocumented (*n* = 39)	p-value
**Hospital course**			
Length of stay, days, median [IQR]	8.0 [6.0–15.0]	7.0 [5.0–11.0]	0.127
Maximal creatinine, mg/dL, median [IQR]	3.3 [2.4–5.0]	2.2 [1.7–3.1]	<0.001
Discharge creatinine, mg/dL, median [IQR]	1.6 [1.1–2.5]	1.5 [1.0–1.8]	0.116
Maximal AKI stage reached during hospitalization by KDIGO creatinine criteria, n (%)			0.015
Stage 1	45 (36.9%)	23 (59.0%)	
Stage 2	21 (17.2%)	8 (20.5%)	
Stage 3	56 (45.9%)	8 (20.5%)	
KRT during hospitalization, n (%)	15 (12.3%)	0 (0.0%)	0.023
**Outcomes**			
In-hospital mortality, n (%)	18 (14.8%)	8 (20.5%)	0.395
Discharge with ongoing KRT, n (%)	9 (7.4%)	0 (0.0%)	0.115
Discharge destination among patients discharged alive, n (%)			–
Home	86 (82.7%)	26 (83.9%)	
Other ward	7 (6.7%)	3 (9.7%)	
Nursing home	10 (9.6%)	2 (6.5%)	
Other hospital	1 (1.0%)	0 (0.0%)	

AKI stage represents the highest creatinine-based KDIGO stage reached during hospitalization and not the stage at first documentation. Discharge destination was summarized among patients discharged alive and was analyzed descriptively because of sparse cell counts; no formal between-group p value is reported. KRT = kidney replacement therapy. A two-sided p-value < 0.05 was considered statistically significant.

The distribution of the maximum creatinine-based AKI stage reached during hospitalization differed between patients with documented and undocumented AKI (overall *p* = 0.015). Undocumented AKI cases more frequently had a maximum stage of 1 compared with documented cases (23/39, 59.0% vs. 45/122, 36.9%), whereas documented cases more frequently reached a maximum stage of 3 (56/122, 45.9% vs. 8/39, 20.5%). Maximum stage 2 occurred at similar rates in the two groups (8/39, 20.5% vs. 21/122, 17.2%). These results describe the highest stage reached during hospitalization and do not indicate the stage at which AKI was first documented.

Length of stay did not differ significantly between groups. KRT during hospitalization occurred only among patients with documented AKI (15/122, 12.3% vs. 0/39, 0.0%; *p* = 0.023). Discharge with ongoing KRT occurred in 9 documented cases (7.4%) and in no undocumented cases. In-hospital mortality was numerically higher in the undocumented group, but did not differ significantly between groups (8/39, 20.5% vs. 18/122, 14.8%; *p* = 0.395). Among patients discharged alive, most patients in both groups were discharged home (82.7% of documented cases and 83.9% of undocumented cases). The remaining destination categories contained small numbers and were summarized descriptively.

### Factors associated with undocumented AKI

In univariable analyses, malignancy was associated with higher odds of undocumented AKI (OR 3.77, 95% CI 1.37–10.32; *p* = 0.010), whereas higher admission and maximal creatinine values were associated with lower odds of undocumented AKI (Table 3).

**Table 3.** Exploratory logistic regression models for factors associated with undocumented AKI.

Predictor	OR for undocumented AKI	95% CI	p-value
**Univariable logistic regression**			
Female sex vs male	1.58	0.76–3.26	0.219
Diabetes mellitus	0.99	0.48–2.03	0.969
Hypertension	0.86	0.38–1.99	0.731
Dyslipidemia	1.33	0.64–2.73	0.445
Congestive heart failure	1.83	0.86–3.94	0.119
Malignancy	3.77	1.37–10.32	0.010
Admission creatinine, per 1 mg/dL	0.68	0.51–0.90	0.006
Maximal creatinine, per 1 mg/dL	0.63	0.47–0.84	0.002
KDIGO stage 2 vs stage 1	0.75	0.29–1.94	0.547
KDIGO stage 3 vs stage 1	0.28	0.11–0.68	0.005
**Primary exploratory multivariable model**			
Malignancy	3.52	1.20–10.32	0.022
Maximal creatinine, per 1 mg/dL	0.64	0.48–0.85	0.002
**Expanded exploratory multivariable model**			
Female sex vs male	1.80	0.79–4.13	0.165
Diabetes mellitus	1.02	0.45–2.32	0.960
Congestive heart failure	1.93	0.81–4.62	0.137
Malignancy	4.12	1.37–12.42	0.012
Maximal creatinine, per 1 mg/dL	0.64	0.48–0.86	0.003

The dependent variable was absence of an explicit documented AKI diagnosis or documented evidence of clinical recognition during hospitalization, with documented AKI as the reference group. ORs above 1 indicate higher odds of undocumented AKI, whereas ORs below 1 indicate lower odds of undocumented AKI. The primary model included 39 undocumented AKI events and two predictor parameters, corresponding to 19.5 events per predictor. The expanded model included five predictor parameters, corresponding to 7.8 events per predictor. The expanded model was considered a secondary robustness analysis. OR = odds ratio; CI = confidence interval; A two-sided p-value < 0.05 was considered statistically significant.

In the primary exploratory multivariable model, which included malignancy and maximal serum creatinine, malignancy remained associated with higher odds of undocumented AKI, whereas higher maximal serum creatinine was associated with lower odds of undocumented AKI. Results were directionally similar in the expanded exploratory model, which additionally included sex, diabetes mellitus, and congestive heart failure (Table 3). KRT was not included in the regression models because it occurred exclusively among patients with documented AKI and resulted in complete separation.

#### Sensitivity analysis restricted to cases with demonstrable creatinine dynamics

A *post hoc* sensitivity analysis was performed in the 149 hospitalizations in which a demonstrable creatinine change could be independently reconstructed from the retained admission, maximal, and discharge serum creatinine values. Of these, 33 cases (22.1%) were classified as undocumented AKI.

In the primary restricted-cohort model, higher maximal serum creatinine remained associated with lower odds of undocumented AKI (adjusted OR 0.67 per 1 mg/dL increase, 95% CI 0.50–0.89; *p* = 0.006), whereas the association between malignancy and undocumented AKI was attenuated and was no longer statistically significant (adjusted OR 2.55, 95% CI 0.79–8.23; *p* = 0.118). Results were similar in the expanded restricted-cohort model, which additionally adjusted for sex, diabetes mellitus, and congestive heart failure (Supplementary Table S1).

Stage 1 AKI remained numerically more frequent among undocumented cases than documented cases (51.5% vs. 34.5%), whereas stage 3 AKI remained less frequent (24.2% vs. 47.4%). However, the overall distribution of maximum AKI stage did not reach statistical significance in the restricted cohort (*p* = 0.058). Between-group differences in length of stay and in-hospital mortality also remained non-significant.

Overall, the association between lower maximal serum creatinine and undocumented AKI was more consistent across analyses, whereas the association with malignancy should be interpreted as exploratory and hypothesis-generating.

Model diagnostics did not indicate problematic multicollinearity, departure from linearity in the logit for maximal serum creatinine, or lack of fit. Cook’s distance identified eight potentially influential observations in the full cohort and seven in the restricted cohort using the 4/n screening threshold. However, leave-one-out analyses did not materially alter the direction or statistical interpretation of the principal estimates.

## Discussion

In this retrospective cohort of patients hospitalized in internal medicine wards, approximately one quarter of serum creatinine-defined AKI cases lacked an explicit diagnosis or documented evidence of clinical recognition. The most consistent finding was the association of underdocumentation with lower admission and maximal serum creatinine values, with the association for maximal creatinine persisting in the sensitivity analysis restricted to hospitalizations in which a demonstrable creatinine change could be reconstructed. In contrast, the association between malignancy and undocumented AKI was attenuated in the restricted cohort and should be regarded as hypothesis-generating rather than as evidence of an established independent association. Importantly, our study extends the evaluation of AKI underdocumentation beyond predominantly critically ill populations to general internal medicine wards. Its principal contribution is the observation that fulfillment of biochemical AKI criteria does not necessarily translate into documentation when absolute creatinine values are lower and may therefore be less clinically salient, suggesting a potential mismatch between criteria based on dynamic kidney-function changes and the way creatinine abnormalities are perceived in routine clinical practice.

The 24.2% underdocumentation rate observed in our cohort was lower than rates previously reported in critically ill populations. Li and colleagues reported unrecognized AKI in 45.8% of older intensive care patients with severe COVID-19, and a subsequent multicenter study reported underdocumentation in 37.2% of older mechanically ventilated patients [10,11]. Esposito et al. also described heterogeneous AKI recognition patterns among hospitalized patients, further supporting the clinical relevance of the gap between laboratory-defined AKI and documented recognition [14]. Direct comparison between studies is limited by differences in patient populations, clinical settings, AKI definitions, and methods used to identify recognition. Our chart-review definition included both explicit diagnostic terminology and documented evidence of clinical recognition rather than relying solely on administrative coding or discharge diagnoses.

Nevertheless, the observed rate should be considered institution specific, as documentation may be influenced by electronic medical record structure, local terminology, physician workload, ward culture, laboratory-monitoring practices, access to nephrology consultation, and discharge-summary requirements. These factors were not measured and may limit generalizability to other hospitals or healthcare systems.

The strongest clinical pattern was the association between the magnitude of serum creatinine elevation and documentation status. Patients with undocumented AKI had lower admission and maximal creatinine values, suggesting that AKI may be more clinically salient when the absolute creatinine concentration is markedly abnormal. However, KDIGO serum creatinine criteria emphasize dynamic change rather than absolute elevation alone [2]. A patient may therefore meet biochemical AKI criteria despite a creatinine value that appears only mildly abnormal in absolute terms. The persistence of the maximal-creatinine association in the restricted sensitivity cohort indicates that this finding was not explained solely by cases in which the AKI event could not be reconstructed from the retained summary measurements. Modest absolute or relative increases in creatinine may therefore fulfill biochemical AKI criteria while remaining less conspicuous to clinicians when the resulting absolute value does not appear markedly abnormal.

Consistent with this finding, undocumented cases more frequently had a maximum AKI stage of 1, whereas documented cases more frequently reached stage 3. However, documentation status reflected recognition at any point during hospitalization. Therefore, these data do not establish that stage 1 AKI was overlooked when it first occurred, because some patients who ultimately reached a higher stage may initially have fulfilled stage 1 criteria and only have been documented after further creatinine elevation. The present data cannot determine the stage at first documentation, quantify delayed recognition, or assess the timeliness of recognition; they indicate only that greater maximum AKI severity was associated with a higher likelihood of documentation during hospitalization.

This finding is clinically relevant because even less severe or transient AKI has been associated with adverse outcomes, including increased mortality, longer hospitalization, and greater resource use [5,7]. The RIFLE literature has demonstrated a graded relationship between AKI severity and mortality, with increased risk evident even at milder stages [8], and transient azotemia has also been associated with increased mortality among hospitalized patients [9]. Thus, underdocumentation of less marked AKI may represent a potential missed opportunity for kidney-related assessment, medication review, monitoring of renal recovery, and discharge planning. Importantly, these care processes were not evaluated in the present study, and no effect of underdocumentation on management or prognosis can be inferred. Interpretation of these findings is also limited by uncertainty regarding baseline kidney function and AKI timing. Although the sensitivity analysis confirmed a demonstrable creatinine change in 149 hospitalizations, it could not determine whether AKI was present at admission or developed during hospitalization; these limitations are discussed further below.

Malignancy was associated with underdocumentation in the full cohort, but this association was attenuated and no longer statistically significant in the restricted sensitivity analysis. Although patients with cancer may experience AKI in multiple clinical contexts, including infection, volume depletion, nephrotoxic treatment, urinary obstruction, metabolic complications, or cancer-related disease processes [15], the small number of affected patients and potential residual confounding preclude attributing the observed association to malignancy itself. This finding should therefore be considered exploratory and requires confirmation in larger cohorts.

The overall distributions of hospitalization indications and retrospectively assigned AKI etiologies did not differ significantly between documentation groups. Because individual categories contained small numbers and only broad hospitalization indications were retained, nominal category-specific differences should be considered descriptive and exploratory.

Pre-renal AKI was the most frequently assigned etiology in both groups, although retrospective etiologic classification may have been subject to misclassification, particularly among undocumented cases in which a formal etiologic assessment may not have been recorded.

No statistically significant differences were observed in length of stay, in-hospital mortality, or discharge destination. The study was not powered to detect modest differences in these outcomes, and the analyses were not designed to establish a causal effect of underdocumentation. KRT occurred only among documented cases, most likely reflecting the greater severity and clinical conspicuity of dialysis-requiring AKI. Such cases generally require specialized evaluation and procedural planning and are therefore unlikely to remain undocumented. AKI requiring KRT may have important implications for renal recovery and long-term outcomes [16], but the observed difference should not be interpreted as evidence that documentation reduced the need for KRT or altered the clinical course.

The potential clinical importance of underdocumentation lies in its possible association with missed kidney-related care processes. Data regarding medication adjustment, nephrotoxin discontinuation or avoidance, repeat creatinine testing, nephrology referral, renal follow-up recommendations, and post-discharge kidney function monitoring were not systematically collected. Consequently, whether undocumented AKI represents a missed opportunity for kidney-related care remains a clinically plausible hypothesis rather than a demonstrated finding. Electronic alerts based on dynamic creatinine changes or other structured AKI detection pathways may represent one approach to reducing underdocumentation, particularly when absolute creatinine values are not markedly elevated. The present study did not evaluate such an intervention. Previous systematic reviews, meta-analyses, and randomized trials suggest that electronic AKI alerts may improve documentation or selected care processes, including medication-related actions, whereas their effects on renal recovery, KRT, mortality, and other patient outcomes remain inconsistent [17–21]. Future prospective studies should therefore assess not only documentation rates but also actionable kidney-related care processes, renal recovery, post-discharge follow-up, and clinical outcomes.

This study has several limitations. First, it was a retrospective, single-center study conducted over six months in internal medicine wards at one hospital. Although the five wards operated under broadly comparable clinical and organizational workflows, physician experience, trainee participation, workload, documentation culture, and nephrology consultation practices may have varied. Ward and treating-team identifiers were not retained; therefore, documentation rates could not be compared across wards, within-team clustering could not be accounted for, and patient-level influences could not be separated from organizational factors, limiting external validity.

Second, AKI was assessed using serum creatinine criteria only because urine-output data were unavailable. Reliable pre-admission creatinine values, exact measurement timestamps, and the number and frequency of creatinine measurements were also not retained. Use of the lower admission or discharge creatinine as the available reference may have resulted in misclassification, particularly among patients with chronic kidney dysfunction, and prevented distinction between admission-associated and hospital-acquired AKI. AKI already present at admission may have been more clinically apparent and more likely to be documented than AKI developing later during hospitalization. Differential laboratory-monitoring intensity between documentation groups may also have influenced AKI detection and cannot be excluded. Although the restricted sensitivity analysis strengthened the principal creatinine finding, it did not resolve these limitations. The findings should therefore be interpreted as applying to serum creatinine-defined AKI identified during hospitalization rather than specifically to hospital-acquired AKI.

Third, documentation status was determined by one primary reviewer using a predefined protocol. Fifteen uncertain cases underwent additional adjudication by a senior nephrologist, but a second reviewer did not independently reassess the entire cohort or a random subset, and formal inter-rater agreement could therefore not be quantified. The definition intentionally included both explicit diagnostic labeling and documented evidence of clinical recognition.

Clinicians may have recognized or acted on a creatinine change without documenting the rationale, whereas the presence of minimal documentation does not establish that AKI assessment, management, or follow-up was complete, appropriate, or timely. Because the specific criterion supporting each positive documentation classification was not separately retained, we could not perform a sensitivity analysis restricted to explicit AKI diagnostic terminology. Accordingly, the reported underdocumentation rate applies to the combined absence of an explicit diagnosis or documented evidence of clinical recognition and may underestimate the absence of formal diagnostic labeling alone.

Fourth, only 39 undocumented AKI events were available, limiting the number of predictors that could be evaluated reliably. The primary model was intentionally parsimonious, whereas the expanded model had a lower event-to-predictor ratio and should be considered a secondary robustness analysis. Model diagnostics did not identify problematic multicollinearity, major departures from linearity, or lack of fit, although several observations exceeded the Cook’s distance screening threshold. Leave-one-out analyses did not materially alter the direction or statistical interpretation of the principal estimates; nevertheless, wide confidence intervals, residual confounding, and model instability cannot be excluded. Interpretation of the malignancy association is additionally limited by the small number of affected patients and the absence of detailed information regarding cancer type and stage, metastatic disease, anticancer treatment exposure, albumin, body mass index, nutritional status, frailty, muscle mass, functional status, illness severity, and goals-of-care decisions. The observed association may therefore reflect reduced creatinine generation, cancer severity, competing clinical priorities, treatment-related factors, or other residual confounding rather than an effect of malignancy itself. Its attenuation in the restricted sensitivity cohort further supports an exploratory interpretation.

Finally, only broad hospitalization-indication categories were retained, and AKI etiology was assigned retrospectively. Specific diagnoses such as acute heart failure, acute coronary syndrome, arrhythmia, cirrhosis or liver failure, and cancer-related complications were not separately collected, and cardiorenal and hepatorenal syndromes were not formally adjudicated.

These features limit diagnosis-specific and mechanistic interpretation. AKI-related care processes, post-discharge renal recovery, recurrent hospitalization, chronic kidney disease progression, and long-term clinical outcomes were also not evaluated. The study therefore cannot determine whether underdocumentation affected management, follow-up, or prognosis, or which specific clinical conditions accounted for the observed category-level patterns.

The study also has several strengths. It examined AKI documentation in general internal medicine wards, a clinically relevant setting that has received less attention than intensive care populations. AKI cases were identified using laboratory data and underwent detailed medical-record review rather than relying exclusively on administrative coding. The documentation definition incorporated both explicit diagnostic terminology and documented evidence of clinical recognition while excluding isolated laboratory abnormalities and nonspecific references to chronic renal dysfunction. In addition, the sensitivity analysis restricted to cases with demonstrable creatinine dynamics supported the robustness of the principal finding. Together, these findings extend the recognized gap between laboratory-defined and documented AKI to general internal medicine wards and identify lower maximal creatinine as a characteristic consistently associated with underdocumentation.

## Conclusion

In this single-center cohort of internal medicine inpatients, approximately one quarter of serum creatinine-defined AKI cases lacked an explicit diagnosis or documented evidence of clinical recognition. Lower maximal creatinine was the most consistent characteristic associated with underdocumentation, whereas the malignancy signal was not robust in sensitivity analysis.

The study did not assess effects on management or outcomes. Prospective multicenter studies should evaluate generalizability and determine whether structured AKI detection and documentation strategies improve kidney-related care processes and clinical outcomes.

## Supplementary Material

Supp Table S1.docx

## Data Availability

The data supporting the findings of this study are not publicly available because they contain information that could compromise participant privacy. De-identified data may be available from the corresponding author upon reasonable request and subject to institutional and ethical approval.
