# Supplementary material for: Underdocumentation of laboratory-defined acute kidney injury in internal medicine wards: a retrospective cohort study
Source: Ren Fail. 2026 Sep 6;48(1):2723684. doi: 10.1080/0886022X.2026.2723684 (PMC13552292; doi:10.1080/0886022X.2026.2723684)
Supplement: Supplementary file 1 [file IRNF_A_2723684_SM0812.docx]

| **Predictor** | **OR for undocumented AKI** | **95% CI** | **p-value** |
| --- | --- | --- | --- |
| **Primary restricted-cohort model** |  |  |  |
| Malignancy | 2.55 | 0.79-8.23 | 0.118 |
| Maximal creatinine, per 1 mg/dL | 0.67 | 0.50-0.89 | 0.006 |
| **Expanded restricted-cohort model** |  |  |  |
| Female sex vs male | 1.49 | 0.63-3.53 | 0.362 |
| Diabetes mellitus | 1.18 | 0.51-2.77 | 0.698 |
| Congestive heart failure | 1.74 | 0.71-4.28 | 0.224 |
| Malignancy | 2.76 | 0.83-9.20 | 0.098 |
| Maximal creatinine, per 1 mg/dL | 0.67 | 0.5-0.9 | 0.008 |

**Supplementary Table S1**. Exploratory logistic regression models for factors associated with undocumented AKI, restricted to hospitalizations with demonstrable creatinine dynamics. The analysis included 149 hospitalizations with a maximal-to-reference serum creatinine difference of at least 0.3 mg/dL and/or a maximal creatinine value at least 1.5 times the available reference. The dependent variable was undocumented AKI, with documented AKI as the reference group. The restricted cohort included 33 undocumented AKI events. The primary and expanded models included two and five predictor parameters, corresponding to 16.5 and 6.6 events per predictor, respectively. ORs above 1 indicate higher odds of undocumented AKI. OR, odds ratio; CI, confidence interval.
